# Influence of Continuous Training on Atrial Myocytes I_K1_ and I_KAch_ and on Induction of Atrial Fibrillation in a Rabbit Model

**DOI:** 10.1155/2018/3795608

**Published:** 2018-12-19

**Authors:** Dou Yuan, Ping Zheng, Chen Tan, Si Hui Huang, Dan Li, Jian Huang

**Affiliations:** ^1^Department of Thoracic and Cardiovascular Surgery, Cheng Du Shang Jin Nan Fu Hospital, West China Hospital of Sichuan University, Chengdu, China; ^2^Clinical Department of Strategic Support Force Aerospace Systems in Beijing Space City, Beijing, China; ^3^Department of Cardiology, HeBei Yan Da Hospital, Langfang, China; ^4^Department of Cardiology, Peking University First Hospital, Beijing, China; ^5^Department of Ultrasound, PLA Army General Hopsital, Beijing, China; ^6^Fuwai Heart Disease Hospital, CAMS and PUMC, Beijing, China

## Abstract

**Background:**

Elucidation of mechanisms underlying continuous training-related atrial fibrillation (AF) may inform formulation of novel therapeutic approaches and training method selection. This study was aimed at assessing mechanisms underlying continuous training-induced AF in an animal model.

**Methods:**

Healthy New Zealand rabbits were divided into three groups (*n*=8 each), namely, control (C), and moderate intensity (M), and high intensity (H) continuous training according to treadmill speed. Atrial size andintrinsic and resting heart rates were measured by transthoracic echocardiography before, and 8 and 12 weeks after training. Using a Langendorff perfusion system, AF was induced by S1S2 stimulation and the induction rate was recorded. Atrial I_K1_ and I_KAch_ ion current densities were recorded using whole-cell patch-clamp technique in isolated atrial myocytes. Changes in atrial Kir2.1, Kir2.2, Kir3.1, and Kir3.4 mRNA expression were assessed by reverse transcriptase-coupled polymerase chain reaction.

**Results:**

After 8 and 12 weeks, Groups M and H vs. Group C had greater (all *P* < 0.05) atrial anteroposterior diameter; greater incidence of AF (60% and 90% vs. 45%, respectively; *P* < 0.05, also between Groups H and M); and greater atrial IK_Ach_ current density. In Group H, Kir2.1 and Kir2.2 mRNA expression in the left and right atria was increased (*P* < 0.05, vs. Groups C and M) as was left atrial Kir3.1 and Kir3.4 mRNA expression (*P* < 0.05, vs. Group C).

**Conclusion:**

In a rabbit model, continuous training enlarges atrial diameter leading to atrial structural and electrical remodeling and increased AF incidence.

## 1. Background

Atrial fibrillation (AF), a common cardiac arrhythmia, is associated with increased risk of stroke, heart failure, and death [1]. Among athletes, AF has an incidence of about 0.2%–0.3%, which is greater than that in the general population [2]. Continuous (vs. interval) training remains common and can induce AF via the autonomic nervous system and atrial structural changes (dilatation and fibrosis) secondary to chronic volume and pressure overload [3]. In the human heart, atrial stretching might result in excessive I_K1_ currents, likely decreasing the effective refractory period, thereby initiating and/or maintaining AF [4]. I_KACh_ is crucial in AF generation as shown in a murine I_KACh_-deficient knockout model [5].

Improved understanding of the mechanisms of continuous training-related AF may help in the development of novel therapeutic approaches [6] and in training method selection. In current study, we designed a rabbit model to test if continuous training can increase AF vulnerability and the potential mechanisms including changes in ion channel currents and gene expression.

## 2. Methods

### 2.1. Ethics Committee Approval

Animal welfare and the relevant experiment were carried out in compliance with the guide for the care and use of laboratory animals. And the experiment was approved by the Ethics Committee of the PLA Army General Hopsital.

### 2.2. Animal Model and Experimental Protocol

**Figure 1:**
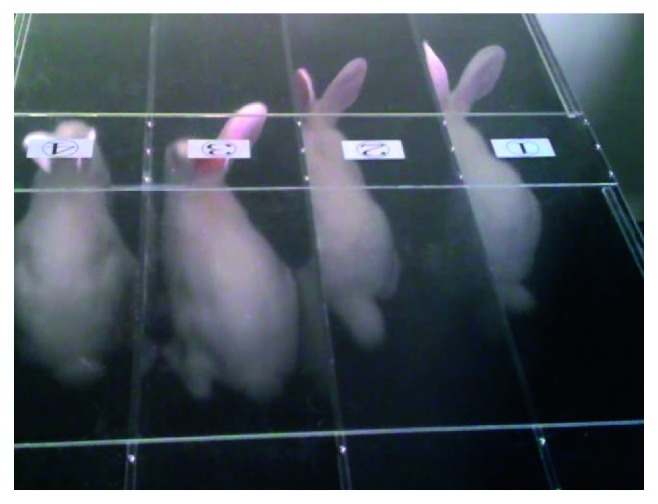
Four rabbits running on the treadmill at the same time.

This study was approved by the institutional animal research ethics committee and conformed to the Guide for the Care and Use of Laboratory Animals. Forty-two pathogen-free New Zealand rabbits weighing 2.5 to 3.0 kg were fed pellet rabbit diet adlibitum and had free access to water. The rabbits were randomly divided into three groups (initially *n* = 14 each, finally *n* = 8 each after eliminating rabbits unwilling to exercise): control(C); and moderate- (M-) and high- (H-) intensity continuous training. Exercise training was performed on a low-speed, levelled, motorized treadmill; the treadmill was fabricated in the Integrated runway and divided into four tracks by using a dummy plate; it was a total volume of 405 L with a total length of 150 cm, height 30 cm, and width 90 cm to keep running the four in sync; it was automatic and adjustable with the angle of 0∼35° and speed of 0∼67 cm/s (the rabbit running platform, which had not been reported so far, was improved based on Gaustad treadmill using a high-powered motor drive and a concurrent four runway operation by Beijing Zhi Bao Biotechnology Co. Ltd. production of rats, Figure 1). The training program was preceded by a 1-week period of adaptation to the treadmill exercise, with 30 min running time and 25 cm/s treadmill speed. Exercise training consisted [7–9] of a 12-week period of running at a speed of 25 cm/s (Group M) and 50 cm/s (Group H) for 60 min (or one-time exhaustive exercise less than 1 h) 5 days per week; all rabbits were tested at inclination ranging 0°; Group C did not do any exercise. Investigators observed the treadmill sessions daily to ensure effective running.

### 2.3. Echocardiography

Transthoracic echocardiographic studies were performed with a phased-array probe 10S (4.5–11.5 Megahertz) in an IE 33 system and under 0.5% sodium pentobarbital anesthesia. Measurements were made at weeks 0, 8, and 12. The atrial anteroposterior diameters were recorded.

### 2.4. Electrophysiological Study

At the end of the study period, all rabbits were anaesthetized and sacrificed with 3% pentobarbital at 30 mg/kg. After midsternal thoracotomy, the heart was rapidly removed and placed in cold perfusion fluid (0°C–10°C). The aorta was cannulated and connected to a Langendorff perfusion system filled with warmed (37°C) Krebs-Henseleit's solution. The composition of the perfusion fluid was (in mM) as follows: NaCl 118, NaHCO_3_ 25, KCl 2.8, CaCl_2_ 2.5, MgSO_4_ 0.5, KH_2_PO_4_ 1.2, Na_2_EDTA 0.57, pyruvic acid 2.0, and glucose 5.5; it was gassed with a mixture of 95% O_2_/5% CO_2_, and pH was kept at 7.35–7.45. The perfusion pressure was maintained at 65 mmHg, with an initial coronary flow of 20 ml/min. To test the inducibility of AF and measure atrial effective refractory periods (AERPs), electrical stimulation was performed with a bipolar electrode attached to the right atrial appendage. The noncontact 12-lead electrocardiogram (ECG) recording system with Wilson terminal was used to record the ECG signals. These signals then were processed using the Biopac ECG amplification system and stored in the computer. ADP90 (the action potential duration of cardiac repolarization of 90%) was automatic analysis with CED Spike II analysis software.

AERPs were measured during sinus rhythm at a S1S2 programmed ectopic stimulation [10, 11]. After an eight-beat train (S1-S1), a single premature stimulus (S2) was delivered. The S1-S2 coupling interval was decreased in 2 ms steps, starting with 120 ms. The shortest S1-S2 interval resulting in a propagated response was defined as an AERP.

**Figure 2:**
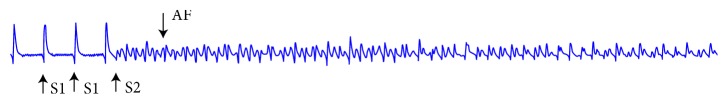
AF was induced by S1S2 incremental stimulation.

Then, AF was induced by S1S2 incremental stimulation (Figure 2), with the cycle length starting from AERPs. Stimuli of 3.3 Hz, 3 ms pulse duration, and threefold diastolic pacing threshold were delivered. The measurement was repeated five times, and episodes of AF were recorded. AF was defined as >1 second of irregular atrial electrograms with irregular ventricular response. Sustained AF (lasting >1 minute) was terminated by bursts.

Following these baseline measurements, one group of pharmacologic agents was added to the perfusate incontinuous infusion for 15–20 minutes and the experiment was repeated. The groups were as follows: acetylcholine (Ach 1 *μ*M/L), Ach+atropine (0.0001, 0.0005, 0.001, and 0.002 mg/ml), and Ach + barium chloride (BaCL_2_ 100 and 500 nM/L and 1, 3, and 6 *μ*M/L).

### 2.5. Patch Clamp Technique

After the electrophysiological study (EPS), the hearts were perfused on a Langendorff apparatus at 37°C by pumping with Tyrode's solution (calcium, 1.8 mmol/L). Following a 5 min perfusion with calcium-free Tyrode's solution, the enzyme solution (50 ml Tyrode's solution with 20 mg type II collagenase and 2 mg trypsin) was perfused for 20–25 min.

When the atrium was loose and translucent, the left atrium and right atrium were sliced into small pieces and placed in Krebs buffer solution containing (mmol/L) KCL 39.97, KH_2_PO_4_ 25, MgSO_4_ 7, H_2_O 3, glucose 10.09, KOH 80, HEPES 10.7, EGTA 0.53, taurine 19.98, and L-glutamic acid 50.3. The cell suspension was filtered through nylon gauze (150 *μ*m mesh), and the cells were stored in the same solution at room temperature for at least 1 h before use. The isolated cells were perfused with extracellular fluid containing *N*-methyl-d-glucamine 149, MgCl_2_ 5, HEPES5, and CaCL_2_ 0.65 (pH 7.4) for 10 minutes.

Only quiescent, rod-shaped cells with clear cross striations were studied. Ionic currents were recorded with whole-cell clamp methods, using an Axopatch 700B amplifier (Axon Instruments). Glass microelectrodes with resistances of 2–3 MΩ were used to record I_K1_. Data were sampled with an A/D converter (Digidata 1322, Axon Instruments) and stored for subsequent analysis. A 3D manipulator (Mp-225, Sutter Instruments) was adjusted to form a stable connection of the electrode tip to the cell surface.

Tip potentials were zeroed before formation of the membrane-pipette seal in tyrode solution. The capacitance and series resistance (Rs) were electrically compensated to minimize the duration of the capacitive surge on the current recording and the voltage drop across the clamped cell membrane. Dof 5 nm/L, 100 *μ*mol/L CdCl_2_, 100 *μ*mol/LTTX, 50 *μ*mmol/L4-aminopyridine (4-AP), and glibenclamide 10 *μ*mol/L were added to the extracellular solution to inhibit current contamination by I_Kr_, L-type Ca^2+^-current, I_Na_, transient outward K^+^-current, and adenosine triphosphate- (ATP-) dependent K^+^-current, respectively. The sampling frequency depended on step stimulation of 10 mV, from −1000 mV to 60 mV, holding potential −40 mV, and clamping time 300 ms, with a frequency of 0.2 kHz used for slowly changing currents.

### 2.6. RNA Isolation and cDNA Synthesis

The atria were snap-frozen in liquid nitrogen and stored at −80°C. Total RNA was isolated with the TRIzol reagent (Invitrogen, USA). RNA was DNase-treated (Fermentas, USA). The quality of isolated RNA was assessed by electrophoresis on polyacrylamide gels (Biowest, Spain).

Briefly, first strand of cDNA (deoxyribose nucleic acid) was synthesized by incubation of 4 *μ*g of RNA specimen in reverse transcription 5 buffer, with 4 *μ*l M-MLVreverse transcriptase (Tresure Biological Engineering (Dalian) Co., Ltd.), 4 *μ*l of T18 primers (China Beijing Taihe Biological Technology Co. Ltd.), 2 *μ*l of dNTP (Tresure Biological Engineering (Dalian) Co., Ltd.), and 1 *μ*l of RNase inhibitor. Synthesis reaction was performed for 5 min at 70°C, 60 min at 42°C, 5 min at 90°C, and 5 min at 4°C.

### 2.7. Semiquantitative Polymerase Chain Reaction (PCR)

The cDNA of interest and the cDNA of the housekeeping gene glyceraldehyde-3-phosphate dehydrogenase (GAPDH) were co-amplified in a single PCR. Primers (China Beijing Taihe Biological Technology Co. Ltd.) were designed for Kir2.1, Kir2.2, Kir3.1, Kir3.4, and GAPDH (Table 1).

The PCR products were separated on agarose gel by electrophoresis and stained with 2 × Ex TaqMix. The density of the PCR products was quantified by densitometry. Linearity for the PCR was established by making a correlation between the number of cycles and the density of gene of interest and GAPDH.

The synthesized cDNA (1 *μ*l) was then used as an amplification template in a 25 *μ*l reaction mixture. After an initial denaturing step at 95°C for 5 minutes, PCR mixes were amplified during 40 cycles with denaturing at 95°C for 30 seconds, annealing at 65°C for 25 seconds, and extension at 72°C for 40 seconds (final extension, 72°C for 7 minutes).

### 2.8. Data Analysis

Group data are expressed as mean ± SE. Statistical analysis was performed with ANOVA. All PCR procedures were performed in duplicate series. One-way or two-way analysis of variance was used for all group comparisons. A *P* value <0.05 was considered statistically significant. SPSS version 19.0 was used for all statistical analyses.

## 3. Results

### 3.1. Structural Remodeling and AF Induction

**Figure 3:**
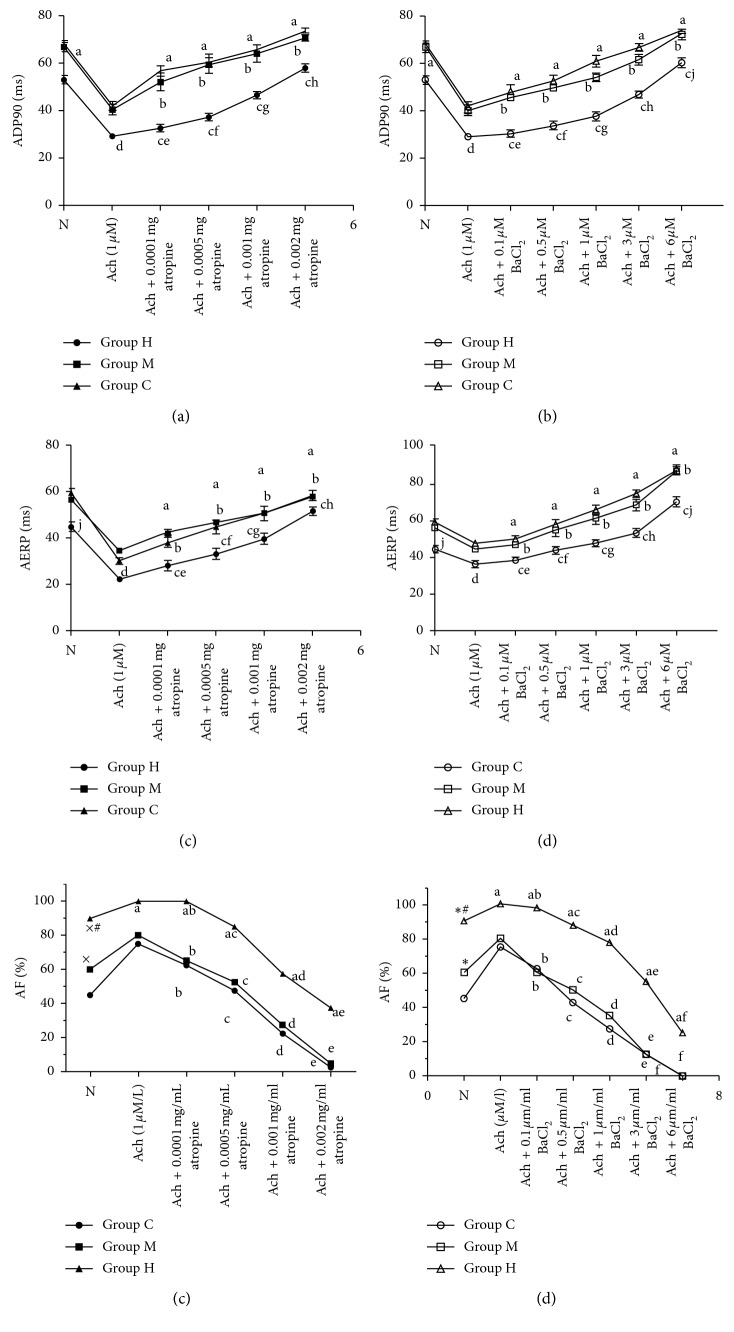
(a) Comparison of atrial APD90 in rabbits with different-intensity continuous training under different Ach + atropine concentrations; (b) comparison of atrial APD90 in rabbits with different-intensity continuous training under different Ach + barium chloride concentrations; (c) comparison of atrial AERP(ms) in rabbits with different-intensity continuous training under different Ach + atropine concentrations; (d) comparison of atrial AERP(ms) in rabbits with different-intensity continuous training under different Ach + barium chloride concentrations. Compared with Groups C and M, ^d^ *P* < 0.05, ^e^ *P* < 0.05, ^f^ *P* < 0.05, ^h^ *P* < 0.05, and ^j^ *P* < 0.05; Compared with Ach alone, ^a^ *P* < 0.05, ^b^ *P* < 0.05, ^c^ *P* < 0.05. (e, f) Comparison of the inductivity of AF in rabbits with different-intensity continuous training under different drug concentrations. (e) Ach + different atropine concentrations; (f) Ach + different barium chloride concentrations. Compared with Group C, ^a^ *P* < 0.05; compared with Ach alone,^b^ *P* < 0.05, ^c^ *P* < 0.05, ^d^ *P* < 0.05, ^e^ *P* < 0.05, and ^f^ *P* < 0.05.

Compared with Group C, the left and right atrial anteroposterior diameters increased in Group M and H after 8 w and 12 w (all *P* < 0.05). Compared with Group M, the left and right atrial anteroposterior diameters increased in Group H after 8 w and 12 w (all *P* < 0.05) (Figure 3). Compared with Group C, the incidence of AF increased in Group M and H (45% vs. 60%, 45% vs. 90%, all *P* < 0.01).

The rabbit atrial APD90 and AERP trend to shorten with training-related is developed increasingly (Groups H vs. Groups C and M, *allP* < 0.05). The ADP90 and AERP prolonged with increasing atropine concentration (0.0001 mg/ml∼0.002 mg/ml) and BaCl_2_ (100 nm∼6M). The ADP90 and AERP were significantly reduced in Group H and were intensity dependent at the same drug concentration (*P* < 0.001). APD90 and AERP were not significantly different between Group M and C (*P* > 0.05). Ach reduced the atrial APD90 and AERP and increased the inducibility of AF. AF could be 100% induced in Group H by Ach, in contrast with 75% and 80% in Groups C and M (*P* < 0.05). The incidence of AF with Ach + atropine (0.002 mg/ml) was 2.5%, 5%, and 37.5% in Groups C, M, and H, respectively (all *P* < 0.05). The incidence of AF with Ach + BaCl_2_ (6 *μ*M) was 0%, 0%, and 25% in Groups C, M, and H, respectively (*P* < 0.05) (Figures 3(a)–3(f)).

### 3.2. Patch Clamping

**Figure 4:**
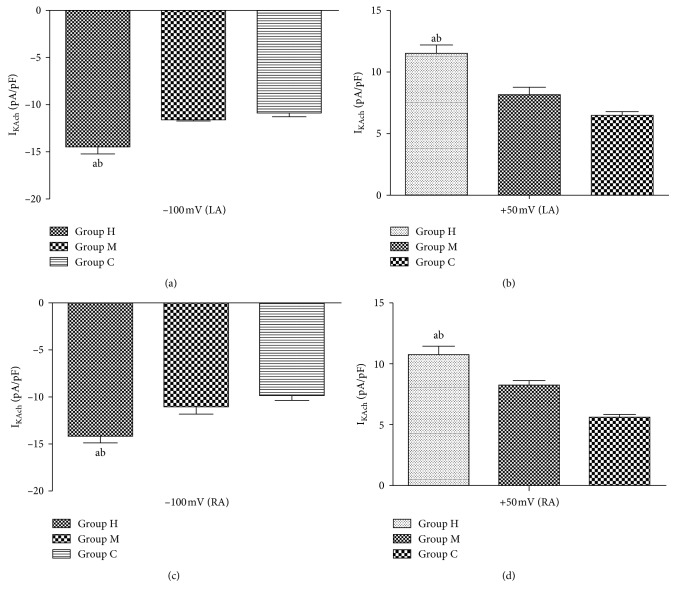
Effects of different-intensity continuous training on atrial tissue IKAch current density in rabbits. (a, c) Clamping voltage: 100 mV; (b, d): clamping voltage +50 mV. Compared with Group C, ^a^ *P* < 0.05; compared with Group M, ^b^ *P* < 0.05.

**Figure 5:**
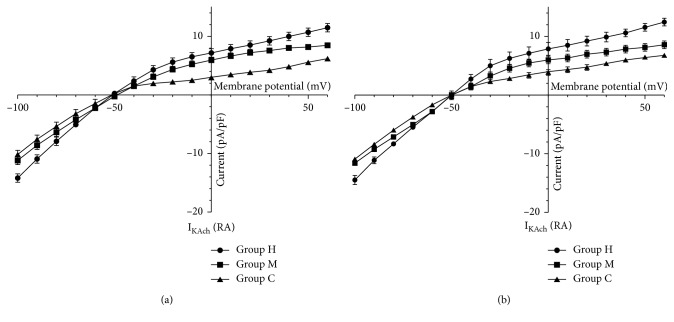
Effects of different-intensity training on atrial tissue IK_Ach_ current-voltage in rabbits. Clamping voltage: 100 mV: (a) RA; (b) LA.

**Figure 6:**
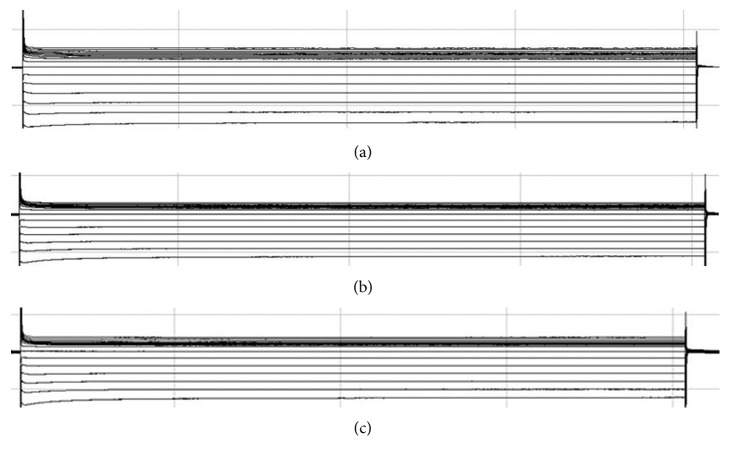
Effects of different-intensity continuous training on atrial tissue IKAch current density in rabbits: (a) Group C; (b) Group M; (c) Group H.

The I_KAch_ current density of left atrial myocytes in Group H was increased (−100 mV: 14.18 ± 1.74 pA/pF; +50 mV: 10.75 ± 1.68 pA/pF), while I_KAch_ current density was 9.92 ± 1.20 pA/pF (–100 mV), 5.57 ± 0.59 pA/pF (+50 mV) in Group C, and 11.07 ± 1.95 pA/pF (−100 mV), 8.25 ± 0.85 pA/pF (+50 mV) in Group M, respectively (Figure 4(a)–4(d), Figure 5(a), 5(b) and Figure 6(a)–6(c)).

The voltage-dependent inward currents could be recorded in both atrial myocytes by I_KAch_ stimulation protocol. I_KAch_ inward current decreased with increasing stimulus pulse. The current density increased when the voltage was more negative in Group H, which was absent in Group M. Reversal potential was about −50mv, above which the current became outward flowing with an obviously outward rectification. The current density of I_KAch_ increased with increasing training intensity, and the current-voltage curve moved up. However, the reversal potential and rectifying properties were not influenced by training intensity (*P* > 0.05).

### 3.3. RT-PCR

**Figure 7:**
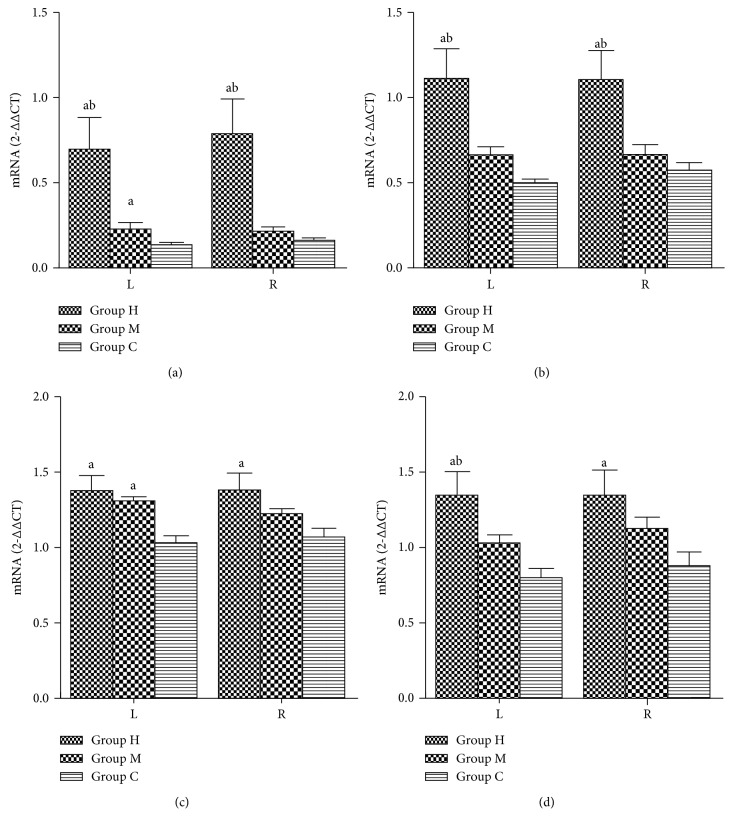
(a–d) Effects of different-intensity exercise training on Kir2.1, Kir2.2, Kir3.1, and Kir3.4 mRNA expression of left and right atria in rabbits. Compared with Group C, ^a^ *P* < 0.05; compared with Group M, ^b^ *P* < 0.05.

Kir2.1, Kir2.2, Kir3.1, and Kir3.4 mRNA expression of the left and right atria as assessed by reverse-transcription polymerase chain reaction (RT-PCR) was higher in Group H relative to Groups C and M (*P* < 0.05). Compared with Group C, Kir2.1 and Kir3.1 mRNA expression in the left atrium was higher in Group M (*P* < 0.05). Compared with Group M, Kir3.4 mRNA expression in the left atrium was higher in Group H (*P* < 0.05) (Figure 7(a)–7(d)).

## 4. Discussion

It is commonly believed that maximal oxygen uptake (VO2max) has been the best measure of heart-lung function in humans [12], which is being used more and more as animal studies [13, 14] to estimate medicinal effect [15]. Some studies [13, 16, 17] have shown that VO2max is connected with inclination of the treadmill at aerobic exercise. Bedford [18] studies have shown intensity training according to oxygen uptake at maximal aerobic exercise: high-intensity at 90% of VO2max and moderate-intensity at 60%∼70% of VO2max; Gaustad [9]studies have shown that all the rabbits' running speed at VO2max was 51 ± 9 cm/s and inclinations of the treadmill was 0–20°, when they looked more comfortable at 0–10° of inclinations ranging. The rabbits were tested at inclination ranging 0°, high-intensity: running speed at 50 cm/s; moderate-intensity: running speed at 25 cm/s in the experimental procedures.

Atrial enlargement is an independent risk factor for AF [19]. This study showed that high-intensity training for 12 weeks could lead to increased atrial diameter and incidence of AF in rabbits, which is consistent with the previous studies [20].

We posit that exercise-induced AF is triggered by left atrial enlargement following increased cardiac preload and ventricular pressure after long-term high-intensity endurance training. Stretch-activated channels of atrial myocytes are activated, and thus ADP90 and AERP are shortened.

K-Ach is a specific potassium channel that is mainly present in atrial myocytes and is activated by acetylcholine released from the vagal nerve. Many studies have shown increased atrial myocyte I_KAch_ current density in patients with AF. In this study, Ach was added into the perfusate, then ADP and AERP were recorded, and AF was induced. The results showed significant shortening of ADP and AERP and inducibility of AF in Group H. Different concentrations (0.0001 mg/ml∼0.002 mg/ml) of the I_KAch_ inhibitor atropine prolonged ADP and AERP and reduced AF inducibility. AF inducibility in Group H was higher than in the remaining two groups at the same concentration of atropine, which suggested that activated I_KAch_ increased the incidence of AF, and inversely, that blocking I_KAch_ reduced the incidence of AF. The latter result is consistent with that of the study by Kovoor [5] et al. Therefore, high-intensity endurance exercise enhanced I_KAch_ response. The increased vagal tone induced by exercise released Ach, which activated receptor type M through G protein, thereby increasing K^+^ outflow and accelerating repolarization, resulting in shortening of APD and ERP.

Atrial myocytes resting potential mainly depends on I_K1_.K^+^ outflow gradually reduced during depolarization, closely relevant with 3rd phase repolarization and ADP [21]. AF inducibility decreased with increasing concentrations (100 nm∼6 M) of the I_K1_ blockerBaCl_2_. At the same BaCl_2_ concentration, AF inducibility in the high-intensity group was higher than in the other two groups, and ADP and AERP were shorter. Therefore, high-intensity endurance exercise enhances I_K1_ response. Tan and her colleagues have confirmed that long-term high-intensity training causes rabbit atrial expansion and increases the inducibility of AF [22].

Subunit compositions of I_K1_ differ among species [23–25]. Human myocardium includes Kir2.1, Kir2.2, and Kir2.3, while only Kir2.1 and Kir2.2 are present in rabbit myocardium. The present study, which is the first to explore the effect of different-intensity endurance exercise on the expression of the K1 subunit in rabbit atrial myocytes, showed that high-intensity continuous training promoted the expression of Kir2.1 and Kir2.2 in rabbit atrial myocytes. Dobrev [26] et al found that I_K1_ increased Kir2.1 mRNA expression in patients with chronic AF. The sympathetic nerve and renin-angiotensin system are activated after exercise, and the adrenergic response acts on protein kinase A (PKA) and C (PKC) [27–29], increasing the Kir2.1 and Kir2.2 expression. K-Ach which is mediated by potassium channels encoded by Kir3.1 and Kir3.4 is mainly present in the mammalian atrium. K-Ach regulates atrial myocytes membrane potential and action potential repolarization and decreases myocardial contractility, excitability, and automaticity.

## 5. Conclusion

The present study showed that long-term high-intensity continuous training increased Kir3.4 and Kir3.1 mRNA expression in the rabbit atrium and I_KAch_ current density.

## Figures and Tables

**Table 1:** Primer information.

Primer name	Sequence (5′ to 3′)	Base number	Product length
GAPDH	F: CAAGTTCCACGGCACGGTCA	20	118 bp
	R: CTCGGCACCAGCATCACCC	19	

Kir2.1	F: TGGTGGTGTTCCAGTCAATC	20	102 bp
	R: CCAGGGTCTCGTTTCTCTTC	20	

Kir2.2	F: GCCAACATGGACGAGAAGTC	20	100 bp
	R: AGGCCAGCGAGAAGATAAGC	20	

Kir3.1	F: CTCTCGGACCTCTTCACCAC	20	114 bp
	R: GATCACCCACCACATGGAC	19	

Kir3.4	F: CTCAGGTCCATCCAAGTCCT	20	104 bp
	R: AGGTGGCAGAGACAACCAAG	20	

## Data Availability

The data used to support the findings of this study are available from the corresponding author upon request.

## Supplementary Materials

Supplementary Materials Supplementary material contains revised articles on parameters of how the treadmill was made; exercise training was performed on a low-speed, levelled, motorized treadmill; the treadmill was fabricated in the Integrated runway by dividing into four tracks by a dummy plate; it was a total volume of 405L with a total length of 150 cm, height 30 cm, and width 90 cm to keep running the four rabbits in sync; it was automatic and adjustable with angle 0–35° and speed 0–67 cm/s (the rabbit running platform, which had not been reported so far, was improved based on the Gaustad treadmill using a high-powered motor drive and a concurrent four runway operation by Beijing Zhi Bao Biotechnology Co. Ltd. production of rats, Figure 1). The training program was preceded by a 1-week period of adaptation to the treadmill exercise, with 30 min running time and 25 cm/s treadmill speed.
