# Supplementary figures and images for: Influence of Continuous Training on Atrial Myocytes IK1 and IKAch and on Induction of Atrial Fibrillation in a Rabbit Model
Source: Cardiol Res Pract. 2018 Dec 19;2018:3795608. doi: 10.1155/2018/3795608 (PMC6313976; doi:10.1155/2018/3795608)

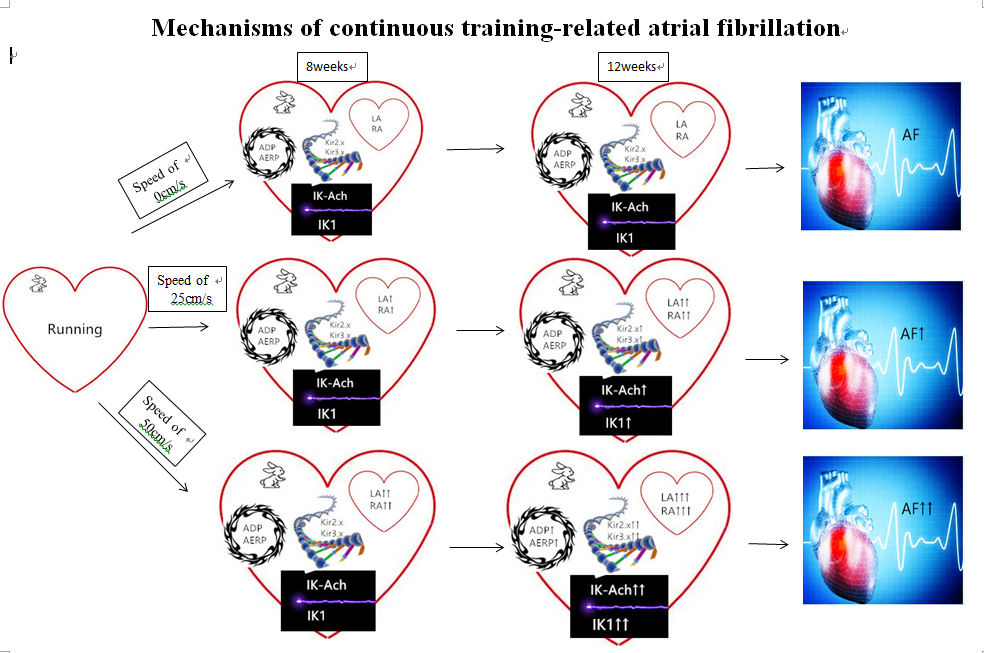

Supplement: Supplementary Materials — Supplementary material contains revised articles on parameters of how the treadmill was made; exercise training was performed on a low-speed, levelled, motorized treadmill; the treadmill was fabricated in the Integrated runway by dividing into four tracks by a dummy plate; it was a total volume of 405L with a total length of 150 cm, height 30 cm, and width 90 cm to keep running the four rabbits in sync; it was automatic and adjustable with angle 0–35° and speed 0–67 cm/s (the rabbit running platform, which had not been reported so far, was improved based on the Gaustad treadmill using a high-powered motor drive and a concurrent four runway operation by Beijing Zhi Bao Biotechnology Co. Ltd. production of rats, Figure 1). The training program was preceded by a 1-week period of adaptation to the treadmill exercise, with 30 min running time and 25 cm/s treadmill speed. [file 3795608.f1.png]
